# Preparation and Performance Evaluation of Antibacterial Melt-Spun Polyurethane Fiber Loaded with Berberine Hydrochloride

**DOI:** 10.3390/polym13142336

**Published:** 2021-07-16

**Authors:** Ruifang Zhao, Pengfei Tan, Yanting Han, Feng Yang, Yidong Shi, Puxin Zhu, Lin Tan

**Affiliations:** 1 College of Biomass Science and Engineering, Sichuan University, Chengdu 610065, China; fangruizhao@126.com (R.Z.); tpf0416@163.com (P.T.); Yangfeng957@163.com (F.Y.); shiyidong@scu.edu.cn (Y.S.); 2 Textile Institute, Sichuan University, Chengdu 610065, China; 3 West China Hospital/West China School of Nursing, Sichuan University, Chengdu 610041, China; yanthan@126.com

**Keywords:** berberine hydrochloride, melt-spun polyurethane fiber, surface coating, antibacterial property

## Abstract

(1) Background: Bacterial infections have long threatened global public safety; hence, it is significant to continuously develop antibacterial fibers that are closely related to people’s daily lives. Berberine hydrochloride is a natural antibacterial agent that has application prospects in the preparation of antibacterial fibers. (2) Methods: This study firstly verified the antibacterial properties of berberine hydrochloride and its possible antibacterial mechanism. Thereafter, berberine hydrochloride was introduced into the self-made melt-spun polyurethane fiber through optimized coating technology. The performance of coating modified polyurethane fiber has been systematically evaluated, including its antibacterial properties, mechanical properties, and surface wettability. (3) Results: Results show that the antibacterial polyurethane fiber with desirable comprehensive properties is expected to be used in the biomedical fields. (4) Conclusions: The research also provides a reference for the development and application of other natural antibacterial ingredients in fiber fields.

## 1. Introduction

Bacterial infection has always been a tough public problem worldwide, which has severely endangered human health and public safety for a long time [1]. A large number of studies have shown that bacteria are the source of transmission of many diseases, such as cholera, tuberculosis, typhoid, and tetanus [2,3]. In order to deal with bacterial infections, various material forms of antibacterial materials have been developed and applied in different fields, such as antibacterial hydrogels, antibacterial fibers, and antibacterial ceramics [4,5]. Among them, antibacterial fiber products have long been widely used in the daily care of special populations, environmental fields, medical systems, and some household textiles [6,7,8,9].

Antibacterial fiber is usually manufactured though introducing an antibacterial agent during the spinning process or after finishing. The commonly used antibacterial agents include silver ions, metal oxides, antibiotics, etc. [10,11]. However, these antibacterial agents have problems such as high cost, physiological toxicity, and drug resistance, and they have encountered certain restrictions in some application fields [12]. Natural medicinal plants have been widely used in Asia area for a long time. They play a vital role in the prevention and treatment of diseases. Therefore, some relatively safe and environmentally friendly natural antibacterial agents extracted from plants or animals have received more and more attention in the preparation of antibacterial fibers [13,14]. Berberine hydrochloride is an isoquinoline alkaloid, which is found in Chinese herbal medicines such as Coptis Chinensis and Cortex Phellodendri. It has broad-spectrum antibacterial activity and low drug resistance, which is widely used to construct antibacterial systems [15,16,17].

Polyurethane (PU) is one of the most widely used polymers. Due to its excellent elasticity, oxygen permeability, biocompatibility, and good processability [18,19,20], PU is widely used in coatings, foams, clothing, and biomedical applications. In the field of large-scale fiber spinning, PU processing methods mainly include melt spinning, wet spinning, and dry spinning. However, dry spinning and wet spinning will inevitably pollute the environment to a certain extent because of the presence of solvents. Melt spinning has a fast production speed and a short process flow, which does not require complex equipment, solvents, and precipitants. Therefore, the application of melt spinning technology will potentially dominate the future production of polyurethane fibers. In addition, since PU fiber is one of the important types of fiber to prepare fabrics, the development of its functionality is particularly urgent. For example, polyurethane fiber can be modified by the aid of environmentally friendly and effective binders (e.g., polyvinyl butyral) combined with functional components (e.g., antibacterial components) through the surface coating process. Compared to the production method wherein the functional groups or components are introduced during spinning, the coating method is more effective, convenient, and low-cost [21,22,23,24].

Herein, this study selected a natural antibacterial component of berberine hydrochloride as the functional component, and its antibacterial properties and antibacterial mechanism were systematically investigated. Afterwards, berberine hydrochloride is introduced into the surface of melt-spun polyurethane fiber by the aid of a binding agent through an optimized coating process. The antibacterial properties, mechanical properties, and surface properties of the fiber are systematically evaluated. The antibacterial polyurethane fiber obtained in this study is expected to be used in the field of biomedical applications as surgical sutures and functional caring fabrics.

## 2. Materials and Methods

### 2.1. Materials

Berberine hydrochloride (BCH), glucose, Tris-HCl buffer, and 2, 3, 5-triphenyltetrazolium chloride were purchased from Chengdu Huaxia Chemical Reagent Co., Ltd. Polyvinyl butyral was provided by Yibin Tianyuan Group Co., Ltd. Absolute ethanol was purchased from Chengdu Kelon Chemical Reagent Factory. Thermoplastic Polyurethane (TPU, TDS-2280A10) was bought from BASF and the polyurethane fiber was prepared by Research Center for Fiber Science and Engineering Technology of Sichuan University through melt spinning technology; *Escherichia coli* (*E. coli*, ATCC 25922) and *Staphylococcus aureus* (*S. aureus*, ATCC6538) were supplied by the R&D Lab of Functional Fibers of Sichuan University. Polyvinyl butyral is industrial grade, and the other reagents are analytical pure.

### 2.2. Minimum Inhibitory Concentration (MIC) of BCH on E. coli and S. aureus

The minimum inhibitory concentration (MIC) values of fibers against bacteria were determined according to the literature with slight changes [25]. Briefly, 100 μL of bacterial mother liquor (*E. coli* and *S. aureus*) was added into 900 μL nutrient broth, which is incubated for 12 h under constant temperature. The BCH aqueous solution with different concentrations were sterilized by UV for 15 min. The activated bacterial solution was diluted to around 10^6^ CFU/mL with sterilized deionized water. Then, 900 μL of BCH aqueous solution was mixed with 100 μL of bacteria solution (*E. coli* and *S. aureus*) in a centrifuge tube. Deionized water with bacteria solution was chosen as a blank control. Samples were incubated in a constant temperature shaker for 12 h, and then 100 μL co-cultured bacterial solution was incubated onto the agar plate under a constant temperature. After 24 h, the agar plates were taken out and the results were recorded. In addition, in order to quantitatively calculate the number of bacteria, the co-cultured bacteria solution was diluted 3–4 times by a 10-fold dilution method and then coated on an agar plate. Similarly, the results were evaluated and recorded after 24 h incubation.

### 2.3. Time-Killing Performance of BCH on E. coli and S. aureus 

In order to investigate the bactericidal effects of BCH, time-kill analysis was curved as Li et al. described [26]. One hundred μL bacterial mother liquor (*E. coli* and *S. aureus*) was added into 900 μL nutrient broth and then incubated under constant temperature for 12 h. The activated bacterial solutions were diluted to ~10^6^ CFU/mL with sterilized deionized water. Then, 100 μL diluted bacterial solution with a concentration of ~10^6^ CFU/mL was mixed with 900 μL BCH solution (the final concentration of BCH is 0.1 mg/mL and 0.3 mg/mL). Next, 100 μL bacterial solution with a concentration of ~10^6^ CFU/mL mixed with 900 μL of deionized water was chosen as a blank control. The samples were incubated under constant temperature by shaker for different intervals (1 h, 2 h, 4 h, 8 h). After that, 100 μL co-cultured bacteria solution was evenly coated on the agar plate and incubated in a constant temperature. After 24 h, the agar plates were taken out and the results are recorded. 

### 2.4. The Effect of BCH on the Respiratory Chain Dehydrogenase Activity of E. coli and S. aureus

The dehydrogenase activity of bacteria was determined by a modified method [27]. Both *E. coli* and *S. aureus* were cultivated to the logarithmic growth phase (~12 h). Then, the bacterial solutions were separately centrifuged (8000 rpm, 3 min) to collect the bacterial pellets. The bacterial pellets were resuspended in deionized water, and 1 mL resuspended bacterial solution, 2 mL of 0.05 mol/L Tris-HCl buffer (pH = 8.6), 2 mL of 0.1 mol/L glucose solution and 2 mL of 1 mg/mL 2, 3, 5-triphenyltetrazolium chloride (red tetrazolium) solution were mixed in a test tube. Next, 1 mL of BCH solution with different concentrations was added to obtain several solutions with the concentrations of 50, 100, 150, 200, and 250 μg/mL. A control group was set up using 1 mL sterilized deionized water instead of BCH solution. The samples were placed in a constant temperature incubator at 37 °C for 5 h. Then, 100 μL concentrated sulfuric acid was added to stop the reaction. Finally, 10 mL of n-butanol was added to each tube for extraction. The supernatants were collected and filtered, and the absorbance of the filtered supernatants was measured at a wavelength of 490 nm (n-butanol is selected as the reference solution). Three parallel experiments are carried out for each group, and the experimental results are averaged.

### 2.5. The Influence of BCH on the Morphology of E. coli and S. aureus

The same procedure was performed to collect the bacterial pellets as above, and then the bacterial pellets were resuspended in deionized water. BCH was added to 5 mL of bacterial suspension (the final concentration of salt BCH was 0.5 mg/mL). Next, 5 mL of the same bacterial suspension without BCH was chosen as control group. The bacterial solutions were cultured in a shaker at 37 °C for 12 h at a speed of 200 r/min. After that, the co-cultured bacterial solution was centrifuged (8000 rpm, 3 min), and the supernatants were discarded to obtain the precipitated bacterial pellets. Next, 10 mL of 2.5% glutaraldehyde aqueous solution was added to fix the bacterial pellets at 4 °C for 12 h. Then, bacterial pellets were dehydrated with 5 mL ethanol with gradient concentrations (0%, 50%, 70%, 90%, and 100%) for 3 min each, and then centrifuged (8000 rpm, 3 min). The bacteria precipitates after dehydration were air-dried naturally. Finally, the morphology of the bacteria was investigated by a scanning electron microscope after sputtering gold plating.

### 2.6. The Effect of BCH on the Release of E. coli Intracellular Macromolecules

The release of cell constituents into the supernatant was examined according to the method described in a previous study [28]. The integrity of cell membranes was evaluated by measuring the leakage of nucleic acids and proteins. *E. coli*, selected as the experimental bacteria, was cultivated to the logarithmic growth phase. Then, the bacterial solution was centrifuged (8000 rpm, 3 min) to collect the bacterial pellet. BCH was added to 200 mL bacterial suspension (~25 μg/mL). The bacterial suspension without BCH was selected as the control group. The bacterial suspension was cultured with shaking in a 37 °C water bath at a shaking speed of 200 rpm/min. In addition, 20 mL bacterial suspension was taken every 1 h, and centrifuged at 8000 rpm for 5 min, 10 mL supernatant was taken, and OD values were measured at 260 nm and 280 nm. Deionized water and 25 μg/mL BCH aqueous solution were used as reference. The experiments were carried out in triplicate, and the experimental results were averaged. 

### 2.7. Preparation and Coating Treatment of Melt-Spun Polyurethane Fiber

Polyurethane pellets were put into an oven and dried with hot air for 12 h to remove the moisture. Then, the dehydrated pallets were transferred into the hopper of the melt spinning machine and extruded by the screw, which was quantified by the metering pump, then formed a bundle of melt-spun fibers through passing the spinneret. After cooling by air, the fibers were oiled with an oil pump. The main components of the oil were dimethyl silicone oil and non-ionic surfactant. After that, the fibers were stretched and collected by a winder to obtain pure melt-spun TPU fiber. Spinning parameters are shown in Table 1 and Table 2.

Polyvinyl butyral (PVB) was dissolved in absolute ethanol to obtain a 5% PVB/ethanol solution. In addition, a certain amount of PVB and berberine hydrochloride (BCH) was weighed and dissolved in absolute ethanol to give a 5% PVB/1% BCH/ethanol solution. The oily agent on the surface of TPU fibers was removed by water. Substantially, TPU fibers were immersed in two coating solutions for 12 h and air-dried naturally. The samples were named as PVB/TPU fibers and PVB/BCH/TPU fibers, respectively. 

### 2.8. Characterizations of Fibers

The surface and cross-sectional morphologies of TPU fibers, PVB/TPU fibers, and PVB/BCH/TPU fibers were observed by SEM (Apreo S HiVoc, Thermo Fisher Scientific). Surface contact angle testers (HARKE-SPCA, Dongguan Shengding Precision Instrument Co., Ltd, USA.) were employed to test the static water contact angle of the TPU fibers, PVB/TPU fibers, and PVB/BCH/TPU fiber samples. Thermal stability of the fibers was determined with thermogravimetric analysis (TGA) (DTG-60 Shimadzu, Japan) under the protection of nitrogen with a heating rate of 10 °C/min from 30 °C to 600 °C. The mechanical properties of the fibers were evaluated by an electronic single yarn strength tester (YM061F0-120 Laizhou Yuanmao Instrument Co., Ltd., China) with a stretching rate of 500 mm/min.

### 2.9. Antibacterial Property of Fibers

The antibacterial property of the prepared fibers against both *E. coli* and *S. aureus* were studied by the oscillation method. First, 40 mg of different fiber samples were washed with deionized water for 30 min to remove impurities such as oil on the surface of the fiber, and then the fiber samples were sterilized by ultraviolet for 15 min in an ultra-clean workbench. Particularly, 100 μL of *E. coli* and *S. aureus* mother liquor was added to 900 μL of liquid culture medium, and placed in a 37 °C constant temperature incubator for 12 h to obtain a saturated bacterial suspension (10^8^~10^9^ CFU/mL). The cultured suspension was diluted 10^3^ times with deionized water, then the sterilized sample was immersed in 1 mL of the diluted bacterial suspension and incubated with shaking for 12 h in a constant temperature water bath shaker at 37 °C. The pure bacterial suspension was selected as a control. Finally, 100 μL liquid was withdrew and evenly coated on the agar plate, which was incubated at 37 °C for 24 h. Then, the results of bacterial growth were recorded.

## 3. Results and Discussion

### 3.1. The MICs and Time-Killing Effect of BCH on E. coli and S. aureus

**Figure 1:**
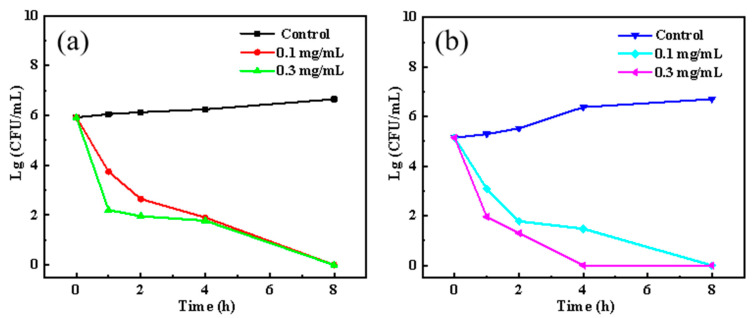
The sterilization curves of BCH on *E. coli* (**a**) and *S. aureus* (**b**).

The antibacterial results of BCH on *E. coli* and *S. aureus* are shown in Table 3. As the concentration of BCH increases, the antibacterial activity of berberine hydrochloride on both bacteria is significantly enhanced. In addition, the MIC of BCH against *S. aureus* is 25 μg/mL and that against *S. aureus* is 50 μg/mL regarding the indicator of a >99% inhibition ratio without bacterial colony. The time-based bactericidal effects of BCH on *E. coli* and *S. aureus* are shown in Figure 1a,b. Both 0.1 mg/mL and 0.3 mg/mL BCH can kill more than 99% *E. coli* bacteria within 1 h, and 8 h are needed to completely inactivate them. Regarding S. aureus bacteria, 0.1 mg/mL BCH can completely inactivate S. aureus within 8 h, while 0.3 mg/mL BCH only needs 4 h to completely inactivate *S. aureus*. Collectively, it can be concluded that the antibacterial activity of BCH against *S. aureus* is slightly better than that against *E. coli*. The explanation should be because BCH is a cationic quaternary ammonium salt with a positive charge, while the surface of *E. coli* and *S. aureus* are all negatively charged and the negative charge on the surface of *S. aureus* is more than that of *E. coli*. Therefore, BCH is more likely to bind to the surface of *S. aureus* based on the electrostatic binding interaction, leading to improved inactivation efficiency.

### 3.2. Measurement of Respiratory Chain Dehydrogenase Activity

**Figure 2:**
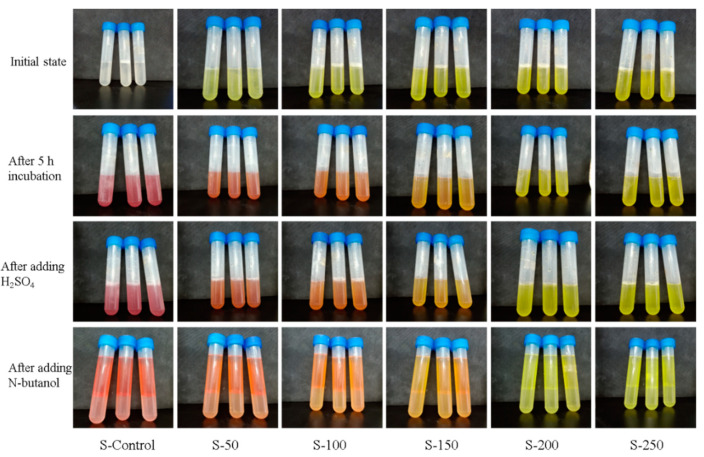
Photographs of *S. aureus* stained with TCC in the control group and BCH-treated groups.

**Figure 3:**
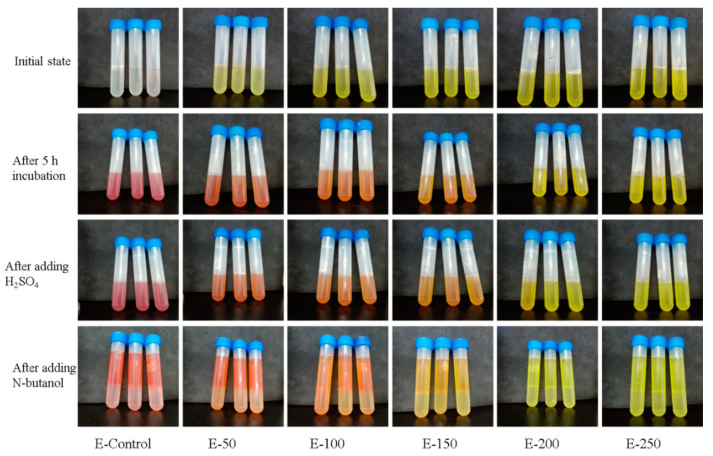
Photographs of *E. coli* stained with TCC in the control group and BCH-treated groups.

**Figure 4:**
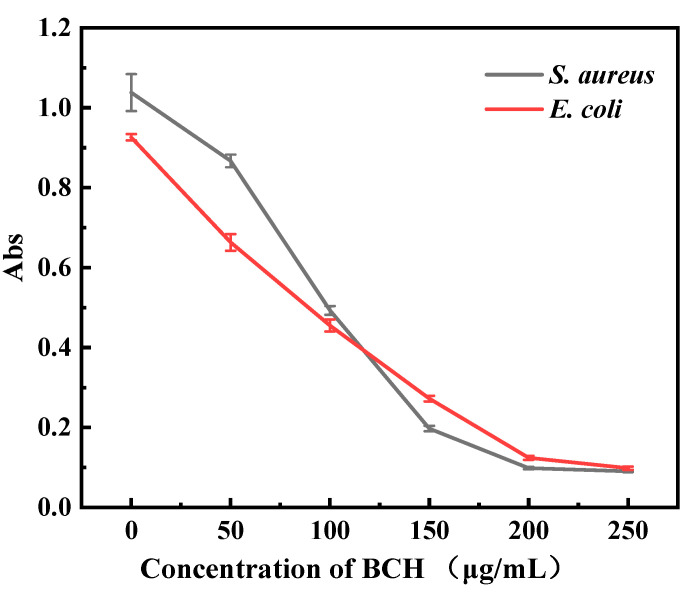
OD values of *E. coli* and *S. aureus* at 490 nm in the BCH-treated groups and the control group.

The lightness of the color indicates a lower the enzymatic activity. As shown in Figure 2 and Figure 3, the color of the control group is the reddest, which indicates the bacterial solution possessing the highest respiratory enzyme activity. As the concentration of BCH increases, the red color of the bacterial solution becomes lighter, indicating that the activity of the respiratory chain dehydrogenase is getting lower. Figure 4 shows that as the concentration of BCH increases, the absorbance value decreases, indicating that the activity of the respiratory chain dehydrogenase in *E. coli* and *S. aureus* cells decreases, which is consistent with the color change of the bacterial solution. Therefore, BCH can significantly depress the activity of respiratory chain dehydrogenase activity of both *E. coli* and *S. aureus*. In addition, with the increase in BCH concentration, the inhibitory effect is more obvious, finally leading to the growth inhibition of *E. coli* and *S. aureus*.

### 3.3. The Influence of BCH on the Morphology of Bacteria

**Figure 5:**
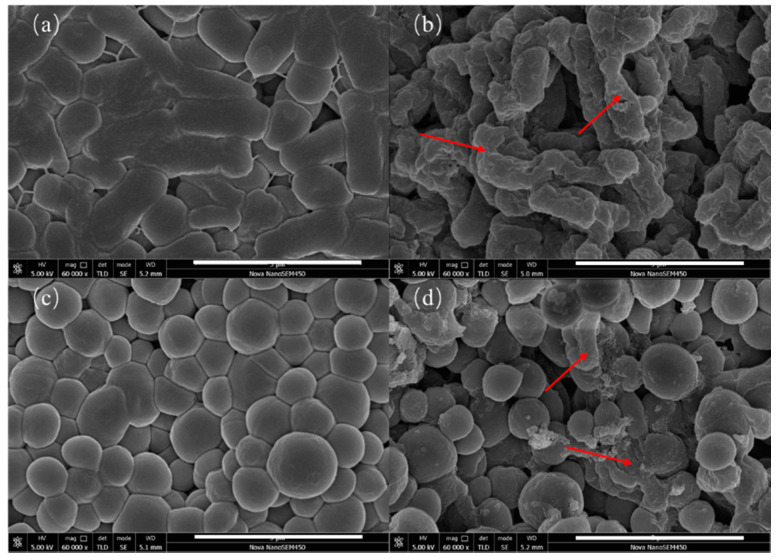
SEM images of control group, *E. coli* (**a**) and *S. aureus* (**c**), and BCH-treated groups, *E. coli* (**b**) and *S. aureus* (**d**).

The SEM images of the control group and BCH-treated *E. coli* and *S. aureus* are shown in Figure 5. It is found that the morphology of both bacteria in the control group were relatively intact, indicating that the bacteria maintain an intact structure without damage. In contrast, the morphologies of BCH-treated *E. coli* and *S. aureus* exhibit obviously contracted and collapsed, indicating that BCH can destroy the cell walls and membranes of *E. coli* and *S. aureus*.

### 3.4. The Effect of BCH on the Release of Intracellular Macromolecules in E. coli

**Figure 6:**
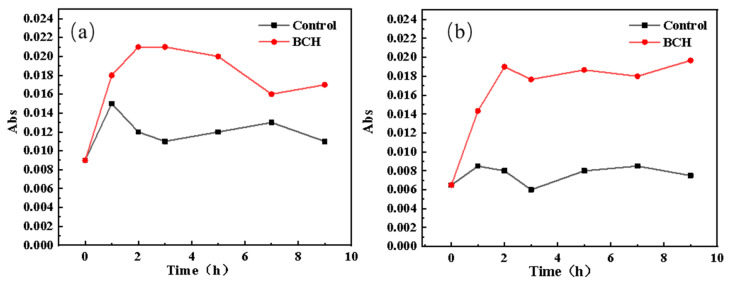
Release curves of *E. coli* protein (**a**) and nucleic acid (**b**).

The release curves of the *E. coli* protein and nucleic acid in the control group and BCH-treated group are shown in Figure 6. Obviously, the release of *E. coli* protein and nucleic acid in the BCH-treated group is significantly higher than that of the control group. Therefore, it is speculated that BCH can change its cell membrane permeability after contacting *E. coli*, and even destroy its cell membrane, so that the intracellular macromolecular substances of *E. coli* leak out.

### 3.5. Fiber Morphologies

**Figure 7:**
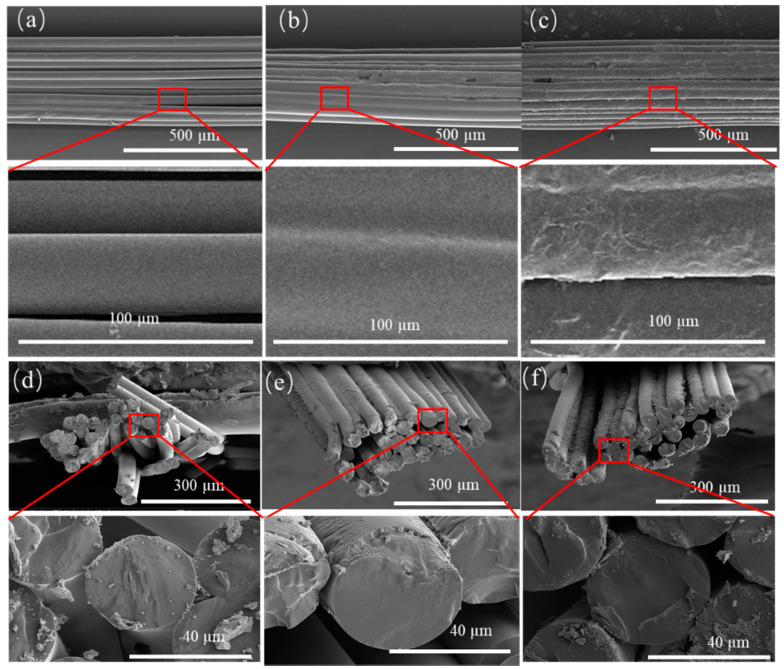
SEM images of the TPU (**a**,**d**), PVB/TPU (**b**,**e**), PVB/BCH/TPU (**c**,**f**) fibers before and after coating.

The morphologies of the TPU fiber, the PVB/TPU fiber, and the PVB/BCH/TPU fiber were investigated by scanning electron microscope (SEM), and the results are shown in Figure 7. the longitudinal sections of the TPU fiber, the PVB/TPU fiber, and the PVB/BCH/TPU fiber can be seen in Figure 7a–c, respectively. It can be seen from the enlarged parts of the SEM images (Figure 7a–c) that the fiber boundaries can be clearly distinguished for the uncoated TPU fiber bundles, while there exist smooth coatings on the surface of the PVB/TPU fiber. Due to such coating film, the border of the fiber becomes inconspicuous. Similarly, the surface of the PVB/BCH/TPU fiber is covered by coatings where the roughness is much larger than that of PVB/TPU fiber. This is due to the irregular film formation after the addition of BCH. Hence, the SEM results can indicate significant differences in the longitudinal cross-sections of different fibers. The cross-sections of the TPU fibers, the PVB/TPU fibers, and the PVB/BCH/TPU fibers are shown in Figure 7d–f. It can be seen from Figure 7d that the uncoated TPU fibers are relatively scattered, and the diameter of a single fiber is about 36 μm. Compared with the PVB/TPU fibers and the PVB/BCH/TPU fibers, the alignment and parallelism between the TPU fibers are lower. After coating, the alignment and parallelism between the fibers as well as the fiber diameter increase.

### 3.6. Surface Wettability Investigation

**Figure 8:**
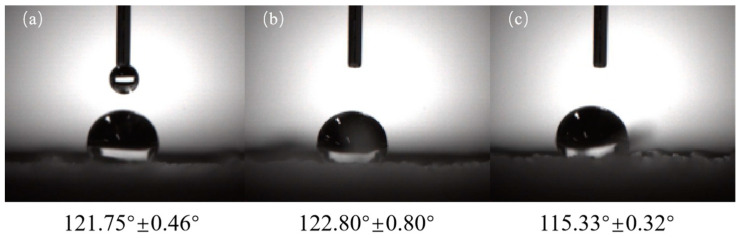
WCAs of the TPU (**a**), PVB/TPU (**b**), and PVB/BCH/TPU (**c**) fibers.

The water contact angle (WCA) of the TPU, PVB/TPU, and PVB/BCH/TPU fibers are shown in Figure 8 and all the WCAs are greater than 90°, indicating that three types of the fibers are hydrophobic. Among them, the TPU fibers are hydrophobic because they contain numerous hydrophobic groups, while the hydrophobicity of the PVB/TPU fiber is a result of the PVB film on the surface. Compared to the PVB/TPU fiber, the hydrophobicity of the PVB/BCH/TPU fiber is relatively smaller. This is because the quaternary ammonium salt structure in BCH is a hydrophilic group, which may reduce the hydrophobicity of the PVB membrane. Additionally, the surface wettability of the fiber will change corresponding to the addition amount. Therefore, antibacterial TPU fibers with different hydrophilic and hydrophobic properties can be obtained by adjusting the concentration of BCH during the preparation process according to the application purposes.

### 3.7. Thermogravimetric Analysis

**Figure 9:**
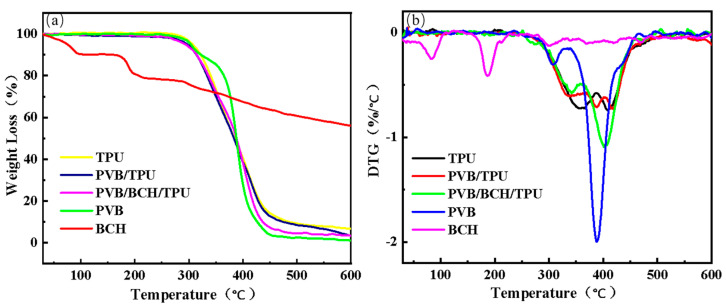
The TG (**a**) and DTG (**b**) curves of different fibers.

The TG (Figure 9a) and DTG (Figure 9b) curves of the melt-spun TPU, PVB/TPU, and PVB/BCH/TPU fibers, and the PVB and BCH are shown in Figure 9. Clearly, the thermal weight loss behavior of the three types of fibers is similar. Although thermal decomposition of PVB is similar to that of the fibers and the first decomposition temperature is lower. In addition, it is noted that BCH has better thermal stability as its two decomposition stages were caused by water evaporation and methoxy cleavage. In addition, the surface coating on the fiber has little effect on the thermal stability.

### 3.8. Mechanical Properties

**Figure 10:**
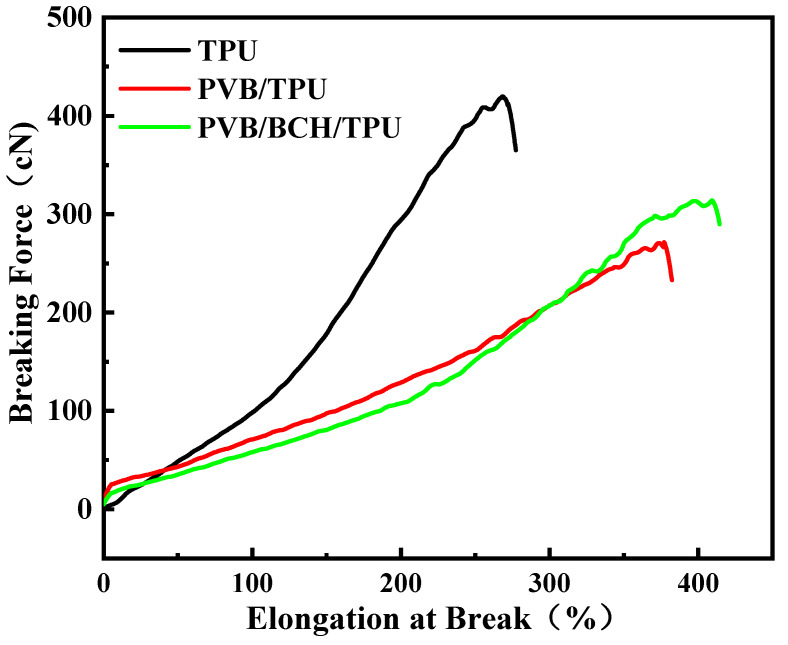
Stress-strain curves of TPU, PVB/TPU, and PVB/BCH/TPU fibers.

The results of mechanical properties of the TPU, PVB/TPU, and PVB/BCH/TPU fibers are shown in Table 4 and Figure 10. The results show that when the surface of TPU fiber is modified by coating, the linear density increases owning to the mass of the film formed on the surface of the TPU fiber increasing its weight per unit length. In addition, the breaking strength of the fiber decreases significantly. This is because the breaking of the fiber and the coating film on the fiber surface are not synchronized with each other during the stretching process. The film breaks first and then the stress is concentrated on the fiber. Compared with pure TPU fibers, the coating-modified fibers reach the fracture threshold earlier. Therefore, the PVB/TPU and PVB/BCH/TPU fibers’ breaking strengths decreased significantly. However, the elongation at break increased. This phenomenon should be further explained by the fact that scattered fibers under asynchronous breaking along with concentrated stress will reduce the elongation at break of the whole bundle of fiber; in contrast, the fibers with a better arrangement degree may exhibit larger breaking elongation in virtue of the uniform distribution of stress during the stretching. Therefore, the breaking elongation of the PVB/TPU and PVB/BCH/TPU fibers are larger than that of the TPU fiber, which may exhibit the application potential in the fields of sportswear and underwear.

### 3.9. Antibacterial Properties of the Fibers

**Figure 11:**
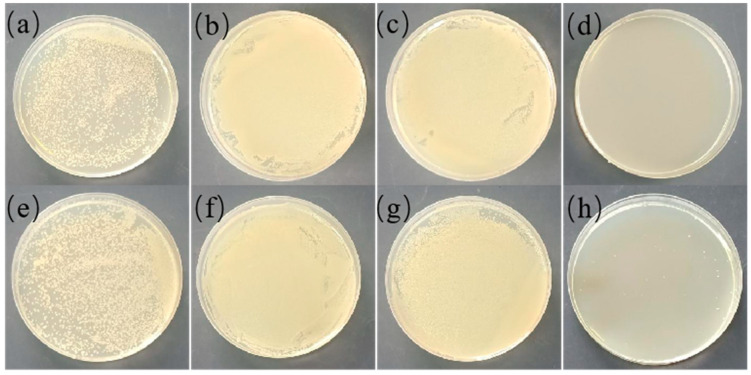
Antibacterial properties of melt-spun TPU fiber against *S. aureus* (**a**) blank control, (**b**) TPU fiber, (**c**) PVB/TPU fiber, (**d**) PVB/BCH/TPU fiber, and against *E. coli* (**e**), blank control, (**f**) TPU fiber, (**g**) PVB/TPU fiber, and (**h**) PVB/BCH/TPU fiber.

The antibacterial results of the TPU, PVB/TPU, and PVB/BCH/TPU fibers are shown in Figure 11. It can be seen that the melt-spun TPU fiber and the PVB/TPU fiber have no antibacterial effect, while obvious antibacterial performance can be observed in the PVB/BCH/TPU fiber groups. In detail, 40 mg PVB/BCH-coated TPU fiber can completely kill *S. aureus* and more than 99.99% of *E. coli* with a concentration of ~10^5^ CFU/mL. Thus, it can be concluded that the introduction of BCH renders the PVB/BCH/TPU fibers with desirable antibacterial properties.

## 4. Conclusions

In this study, we systematically studied the antibacterial activity and mechanism of natural BCH, and successfully introduced it into the surface of polyurethane fibers through a simple coating technology by using PVB as the binder. The antibacterial properties of the yielded fiber can be achieved by depressing the activity of respiratory chain hydrogenase, thus inhibiting the growth of bacteria and, finally, killing them. Additionally, it can destroy the cell wall and cell membrane of the bacteria, causing the leakage of intracellular macromolecules, which can also induce the death of the bacteria. The introduction of BCH gives the polyurethane fiber good antibacterial properties, enhanced breaking elongation, and desirable surface wettability. Therefore, the antibacterial polyurethane fiber prepared in this study is expected to be used in the biomedical fields.

## Figures and Tables

**Table 1:** Temperature parameters during melt spinning.

Section	Zone-1	Zone-2	Zone-3	Zone-4
Temperature/°C	120	190	195	195
Section	Extrusion head	Metering pumps	Pump base	Melt pipeline
Speed	200 m/min	205 m/min	205 m/min	205 m/min
Temperature/°C	200	195	195	195

**Table 2:** Melt spinning speed.

Section	Metering Pump A	Metering Pump B	Oil Pump	Roller 1
Speed	15 r/min	15 r/min	12 r/min	200 m/min
Section	Roller 2	Roller 3	Roller 4	Winder
Speed	200 m/min	205 m/min	205 m/min	200 m/min

**Table 3:** Antibacterial results using different concentrations of BCH.

	Strains	Concentrations of Berberine Hydrochloride (μg/mL)				
		6.25	12.5	25	50	100
Inhibition ratio (%)	*E. coli*	28.2609	63.7681	92.0290	99.9997	100
	*S. aureus*	93.6800	96.8400	99.9973	100	100

**Table 4:** Mechanical properties of TPU, PVB/TPU, and PVB/BCH/TPU fibers.

Fiber Type	Linear Density (Tex)	Breaking Strength (cN/Tex)	Elongation at Break (%)
TPU	34.90 ± 1.25	12.13 ± 0.40	262.47 ± 6.99
TPU/PVB	53.15 ± 0.54	5.19 ± 0.27	366.21 ± 19.88
TPU/PVB/BCH	54.59 ± 0.44	5.81 ± 0.29	403.06 ± 17.98

## Data Availability

The study did not report any data.
